# Taurine and betaine promote lactate clearance in canine hepatocytes by enhancing gluconeogenesis

**DOI:** 10.3389/fvets.2026.1725980

**Published:** 2026-04-13

**Authors:** Yan Ma, Jiaxi Li, Yunliang Li, Lei Lv

**Affiliations:** 1 Nourse Centre for Pet Nutrition, Wuhu, China; 2 Shanghai Chowsing Pet Products Co., Ltd., Shanghai, China; 3 Wuhu Weishi Biotechnology Co., Ltd., Wuhu, China; 4 School of Food and Biological Engineering, Jiangsu University, Zhenjiang, China

**Keywords:** betaine, canine, intense exercise, lactate accumulation, taurine

## Abstract

While lactate accumulation in muscles contributes to fatigue, its efficient clearance-primarily *via* hepatic gluconeogenesis (Cori cycle)-is vital for recovery and sustained performance. This study investigates whether taurine and betaine can enhance this hepatic lactate clearance capacity by modulating gluconeogenesis in canine hepatocytes. The results showed that taurine and betaine significantly promoted lactate clearance in canine hepatocytes. In addition, taurine and betaine significantly increased the levels of phosphoenolpyruvate and glucose in hepatocytes. Meanwhile, taurine and betaine increased the mRNA and protein expression levels of gluconeogenesis-related gene, including pyruvate carboxylase (PC), phosphoenolpyruvate carboxykinase (PEPCK), fructose-1,6-bisphosphatase (FBP1) and glucose-6-phosphatase (G6PC). Consistently, the enhanced activities of PEPCK and G6PC provide functional evidence that taurine and betaine boost the gluconeogenic flux in hepatocytes. Taken together, this study reveals the potential regulatory effects of taurine and betaine on lactate metabolism and gluconeogenesis in canine hepatocytes, providing new insights for recovery strategies in canines after exercise.

## Introduction

Post-exercise recovery in canines aims to facilitate rapid return to normal physiological states following intense exercise. Effective recovery involves not only muscle restoration (1) but also energy replenishment (2), acid–base balance, immune function (3), and oxidative stress response (4). Recovery efficiency determines a canine’s ability to adapt to exercise loads and reduce injury risk (5). Notably, during intense exercise, canine muscle cells may undergo anaerobic metabolism due to hypoxia, leading to the production of lactic acid (6). Excessive lactate accumulation causes muscle fatigue, impairs contraction, and may induce blood acidification, reducing performance.

Taurine, a natural antioxidant, reduces exercise-induced oxidative stress (7, 8). Studies show taurine enhances post-exercise recovery by decreasing intracellular free radicals (9), reducing inflammatory responses (10), and improving the stability of cell membranes (11). Furthermore, it plays a role in regulating amino acid metabolism (12) and energy metabolism (8) in muscles following exercise, thereby enhancing the efficiency of muscle repair post-exercise. Similarly, betaine supplementation reduces muscle soreness and damage (13), lowers inflammatory cytokines (14) and muscle damage markers (15), and enhances protein synthesis via methyl donation, supporting muscle repair (16).

The regulation of hepatic gluconeogenesis by betaine and taurine has gained increasing attention. Betaine supplementation ameliorates fatty liver and improves insulin sensitivity—processes linked to gluconeogenic dysregulation (17). Notably, betaine has been reported to influence the expression of key gluconeogenic enzymes, such as phosphoenolpyruvate carboxykinase (PEPCK) or glucose-6-phosphatase (G6PC) (18, 19). Taurine’s role is more multifaceted, involving calcium modulation, membrane stabilization, and antioxidant activity. Transcriptomic analyses show taurine alters expression profiles of genes involved in amino acid and carbohydrate metabolism in hepatocytes (20). Classical biochemical studies identified taurine as a regulator of pyruvate dehydrogenase (PDH) phosphorylation, a pivotal node determining substrate flux between oxidation and gluconeogenesis (21). Furthermore, recent *in vivo* exercise studies link taurine supplementation to enhanced endurance via delayed hypoglycemia, implying a supportive role in hepatic glucose production during metabolic stress (22, 23). However, a systematic comparison of how taurine and betaine directly modulate gluconeogenic flux and lactate metabolism during post-exercise recovery in canines is lacking.

In this study, we investigated the effects of taurine and betaine on lactate metabolism and gluconeogenic activity in primary canine hepatocytes. Our findings demonstrate that both taurine and betaine significantly enhance lactate clearance, elevate intracellular levels of phosphoenolpyruvate and glucose, and upregulate the expression of key gluconeogenic genes. This study provides mechanistic insights into how taurine and betaine reduce lactate accumulation by enhancing gluconeogenesis, highlighting their potential significance in managing lactate homeostasis in canines.

## Materials and methods

### Cell culture

In this study, we used the canine liver cell lines PETCC2590 and PETCC2593 were obtained from the PETCC (Nourse, Wuhu, China). These immortalized cell lines were originally derived from healthy canine liver tissue and have been characterized to retain key hepatocyte metabolic functions, including gluconeogenic capacity, glycogen storage, and responsiveness to hormonal stimuli (e.g., insulin and glucagon), making them a suitable *in vitro* model for investigating hepatic metabolic pathways. All experiments were performed with cells between passages 5 and 20 to ensure phenotypic stability. Cells were cultured in Dulbecco’s Modified Eagle Medium (DMEM) supplemented with 10% fetal bovine serum (FBS) and 1% penicillin/streptomycin (P/S). Cells were maintained at 37 °C in a humidified atmosphere of 5% CO₂. Cells were seeded at a density of 1.0 × 10^5^ cells/cm^2^ in standard polystyrene tissue culture-treated plates/dishes (Jetbiofil, Guangzhou, China). Treatments with taurine, betaine, or vehicle were initiated 24 h post-seeding, once cells had reached approximately 70–80% confluence and were in a stable, adherent state. Treatment durations are specified for each assay (24 h for gene expression, 12 h for metabolite analysis).

### Reagents and suppliers

Taurine: Macklin, Cat. No. T6017, purity ≥99%. Betaine: Macklin, Cat. No. B802317, purity ≥98%. DMEM base medium: Meilunbio, Cat. No. MA0212. L-Lactic acid sodium: MCE, Cat. No. HY-W040233, purity ≥98%. Fetal Bovine Serum (FBS): Gibco, Cat. No. 10270106, heat-inactivated.

### Cytotoxicity assessment

Prior to all functional assays, we performed cytotoxicity tests using the CCK-8 assay (Meilunbio, Cat. No. MA0218). Cells were treated with the full range of concentrations of taurine and betaine used in the study for 24–72 h. No significant reduction in cell viability was observed at any concentration or time point used in our main experiments, confirming that the reported metabolic effects are not secondary to cytotoxicity.

### Detection of cellular glucose and phosphoenolpyruvate levels

Following the designated treatment, the culture medium was immediately and completely aspirated. To rapidly quench metabolism and remove extracellular metabolites, the cell monolayer was washed three times with cold (4 °C) and sterile Phosphate-Buffered Saline (PBS). Each wash was performed by gently adding pre-chilled PBS, rocking the dish, and thoroughly aspirating within 15 s to minimize metabolic perturbation. Immediately after the final wash, the extraction solution was added directly to the adherent cells on the culture dish by using the assay kit. The resulting extract was then collected for subsequent metabolite analysis. All aforementioned procedures were performed on ice. Cellular glucose levels were quantified using the Glucose-Glo™ Assay Kit (Promega, Cat No. J6021) and phosphoenolpyruvate levels were quantified using the phosphoenolpyruvate Assay Kit (Coibo Cat No. CB10095-Bt) according to the manufacturer’s protocol.

### Detection of lactate generation

At the designated time points, aliquots of cell culture medium (100 μL) were collected directly from each well. Samples were immediately centrifuged at 3,000 × g for 5 min at 4 °C. And then the resulting cell-free supernatant was then transferred to a new tube for the following analysis. Lactate concentration was quantified using a commercial assay kit (Mmbio, Cat No. ADS-F-T009-48) according to the manufacturer’s protocol. The measured concentrations were normalized to the total cellular protein content.

### Reverse transcription and real-time quantitative PCR assay

RNA was extracted from cells harvested from one well of a 6-well plate by using the EZBioscience RNA purification kit. 1 μg of total RNA was used for the cDNA synthesis reaction. The quality of all RNA samples was evaluated by detecting A260/A280 and A260/A230 ratios. Measured using a NanoDrop spectrophotometer. All samples had A260/A280 ratios between 1.9 and 2.1 and A260/A230 ratios > 2.0, indicating high purity free of protein and organic solvent contamination. Reverse transcription was performed with EZBioscience Reverse Transcription Master Mix. In this study, our results demonstrated that beta-actin exhibits high stability across our experimental conditions, indicating its potential as a reliable endogenous control gene. Quantitative real-time PCR (qRT-PCR) was conducted using the Applied Biosystems 7,300 Plus system, with *β*-actin serving as the endogenous control. Relative gene expression was calculated using the 2^−ΔΔCT^ method, with primer sequences provided in Table 1.

**Table 1:** Primer sequence of gluconeogenesis-related gene.

Gene name	Primer	Sequence (5′ → 3′)	GenBank accession no.	Product size (bp)
PC	Forward	GTGGCCAACAGAGGTGAGAT	XM_038424843.1	125 bp
	Reverse	TTCATCTGCTTTCTGCCGGT		
PEPCK	Forward	CTAGCAGGAAGAACCGCGAA	XM_038432791.1	86 bp
	Reverse	CGGACACCAAGGCTTCCTAC		
FBP1	Forward	GATATCGTCACCCTGACCCG	XM_038654998.1	156 bp
	Reverse	AGCAATCCCATAGAGGTGCG		
G6PC	Forward	AGCCTTCTCAAGAACGTGGG	NM_001419280.1	80 bp
	Reverse	CCCTTGCAGCTTTCCCTGTA		

### Western blot

Cell lysates were separated by SDS-PAGE and transferred to PVDF membranes. In this work, we used the homemade SDS-PAGE gels, and all raw materials were obtained from Epizyme Biotech. Phosphate-Buffered Saline with Tween-20 (PBST: 137 mM NaCl, 2.7 mM KCl, 10 mM Na₂HPO₄, 1.8 mM KH₂PO₄, 0.1% Tween-20.) for membrane washing. Membranes were blocked by using 5% skim milk prepared in PBST for 1 h at room temperature with gentle agitation. The membranes were probed overnight at 4 °C with primary antibodies against: FBP1 (12842-1-AP, Proteintech, 1:5000), PC (16588-1-AP, Proteintech, 1:2,000), G6PC (29084-1-AP, Proteintech, 1:5,000), PEPCK (14892-1-AP, Proteintech, 1:2000), or *α*-tubulin (14555-1-AP, Proteintech, 1:1500). After washing with PBST, membranes were incubated with horseradish peroxidase-conjugated secondary antibodies (1:5000) for 1 h at room temperature. Protein bands were visualized using enhanced chemiluminescence.

### Phosphoenolpyruvate carboxykinase and glucose-6-phosphatase activity assay

Following the designated treatments, cells were washed twice with cold PBS and lysed directly on the culture plate. The lysate was collected and subjected to centrifugation at 12,000 × g for 15 min at 4 °C. The supernatant was collected and used for further experiments. PEPCK activity was measured using a commercial colorimetric assay kit (Solarbio, Cat# BC3315) according to the manufacturer’s instructions. G6PC activity was determined using a commercial glucose-6- phosphatase activity assay kit (Solarbio, Cat# BC3325). The total protein concentration of each sample was determined using a BCA Protein Assay Kit (Beyotime, Cat# P0010) to normalize enzymatic activity.

### Statistical analysis

Data are presented as mean ± SEM from three independent experiments. Statistical analyses were performed using GraphPad Prism 9.0 (GraphPad Software). Data between two groups were compared using the Student’s *t*-test. Followed by *post hoc* tests where appropriate. To complement significance testing, effect sizes were calculated for all comparisons. For Student’s *t*-test, Cohen’s d was used. Effect sizes were interpreted as small (0.2 ≤ d < 0.5), medium (0.5 ≤ d < 0.8), and large (d ≥ 0.8). Significance levels are denoted as follows: * *p* < 0.05, ** *p* < 0.01, *** *p* < 0.001, and **** *p* < 0.0001; ns indicates not significant.

## Results

### Taurine and betaine promote lactate clearance in canine hepatocytes

**Figure 1:**
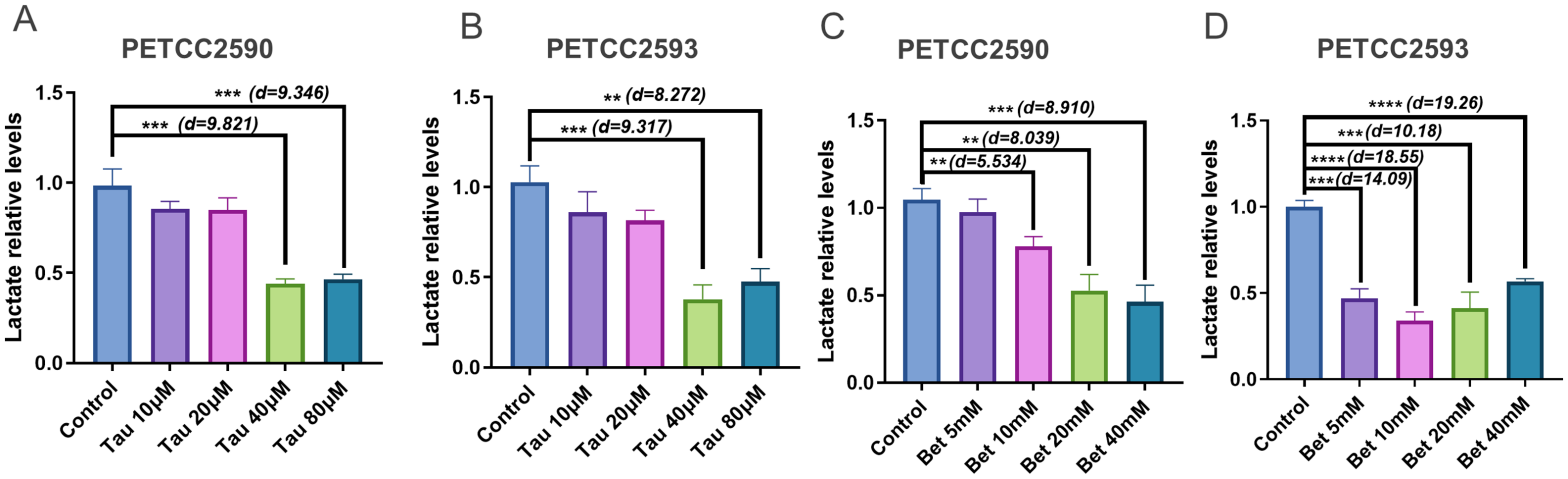
Taurine and betaine promote lactate clearance in canine hepatocytes. Canine hepatocytes were treated with varying concentrations of taurine **(A,B)** and betaine **(C,D)** for 48 h, and the lactate levels was measured. Values are means ± SD from *n* = 3 independent experiments. Statistical differences were determined by Student’s *t* test. * *p* < 0.05, ** *p* < 0.01, *** *p* < 0.001, **** *p* < 0.0001. NS, no significance. Effect sizes (Cohen’s d) for significant comparisons are provided in figure.

In this study, we selected canine hepatocyte cell lines PETCC2590 and PETCC2593 as the cellular models. Specifically, we first pretreated cells with sodium lactate in the medium to mimic the lactate environment. And then cells were treated with taurine or betaine for 12 h prior to medium collection for lactate assay. As shown in Figure 1, treatment with 40 μM (PETCC2590: Student’s *t*-test, df = 4, *p* = 0.0006, Cohen’s d = 9.821) (PETCC2593: Student’s *t*-test, df = 4, *p* = 0.0007, Cohen’s d = 9.317) and 80 μM (PETCC2590: Student’s *t*-test, df = 4, *p* = 0.0009, Cohen’s d = 9.346) (PETCC2593: Student’s *t*-test, df = 4, *p* = 0.0012, Cohen’s d = 8.272) taurine markedly reduced lactate levels in both cell lines (Figures 1A,B). Similarly, we applied different concentrations of betaine to PETCC2590 and PETCC2593 cells, and the findings revealed that in PETCC2590 cells, lactate levels were significantly decreased after treatment with betaine concentrations exceeding 10 mM (PETCC2590: Student’s *t*-test, df = 4, *p* = 0.0052, Cohen’s d = 5.534) (Figure 1C). However, in PETCC2593 cells, a treatment with 5 mM of betaine resulted in a significant reduction in lactate levels (PETCC2593: Student’s *t*-test, df = 4, *p* = 0.0001, Cohen’s d = 14.09) (Figure 1D).

### Taurine and betaine increase phosphoenolpyruvate levels in canine hepatocytes

**Figure 2:**
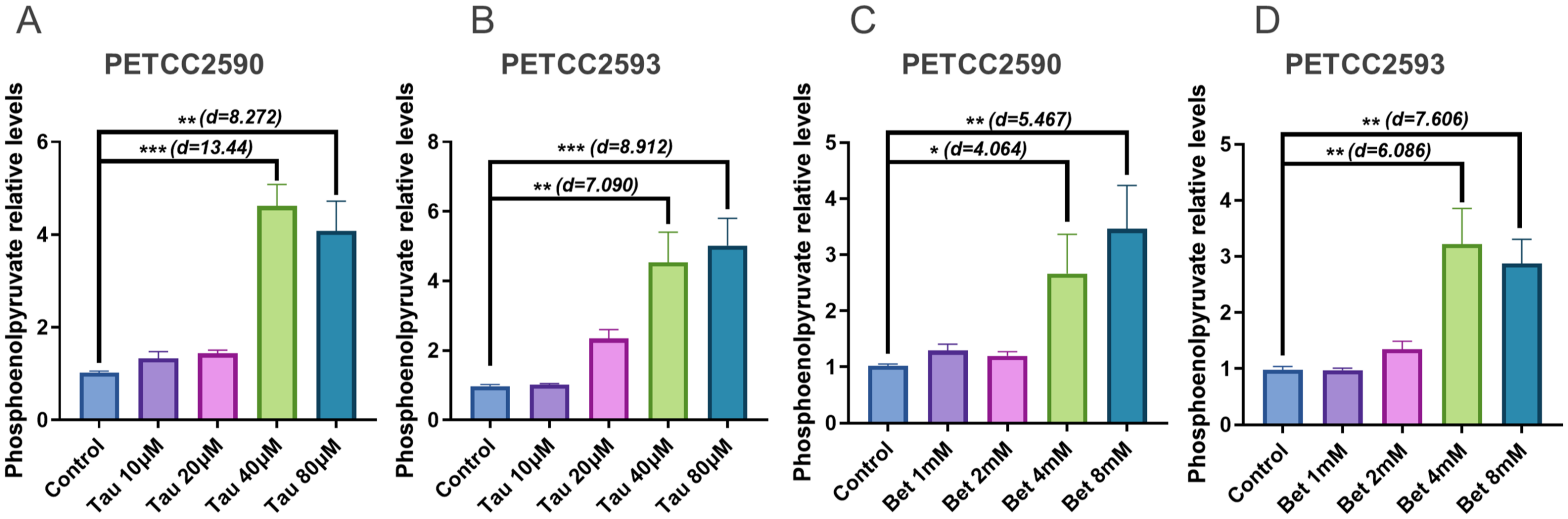
Taurine and betaine increase phosphoenolpyruvate levels in canine hepatocytes. Canine hepatocytes were treated with varying concentrations of taurine **(A,B)** and betaine **(C,D)** for 48 h, and the phosphoenolpyruvate levels was measured. Values are means ± SD from *n* = 3 independent experiments. Statistical differences were determined by Student’s *t* test. * *p* < 0.05, ** *p* < 0.01, *** *p* < 0.001, **** *p* < 0.0001. NS, no significance. Effect sizes (Cohen’s d) for significant comparisons are provided in figure.

Phosphoenolpyruvate serves as a critical intermediate in gluconeogenesis, linking glycolysis-derived substrates to glucose synthesis (24). Subsequently, we measured the levels of phosphoenolpyruvate in canine hepatocyte cell lines PETCC2590 and PETCC2593 following treatment with taurine and betaine. The results indicated that treatment with 40 μM (PETCC2590: Student’s *t*-test, df = 4, *p* = 0.0002, Cohen’s d = 13.44) (PETCC2593: Student’s *t*-test, df = 4, *p* = 0.0021, Cohen’s d = 7.090) and 80 μM (PETCC2590: Student’s *t*-test, df = 4, *p* = 0.0012, Cohen’s d = 8.272) (PETCC2593: Student’s *t*-test, df = 4, *p* = 0.0007, Cohen’s d = 9.317) taurine led to an increase in phosphoenolpyruvate levels (Figures 2A,B). Similarly, treatment with 4 mM (PETCC2590: Student’s *t*-test, df = 4, *p* = 0.0153, Cohen’s d = 4.064) (PETCC2593: Student’s *t*-test, df = 4, *p* = 0.0037, Cohen’s d = 6.086) and 8 mM (PETCC2590: Student’s *t*-test, df = 4, *p* = 0.0054, Cohen’s d = 5.467) (PETCC2593: Student’s *t*-test, df = 4, *p* = 0.0016, Cohen’s d = 7.606) betaine also resulted in an increase in phosphoenolpyruvate levels (Figures 2C,D).

### Taurine and betaine increase glucose secretion levels by enhancing gluconeogenic flux in canine hepatocytes

Quantifying glucose levels is essential to validate whether the observed reduction in lactate secretion results from enhanced gluconeogenic conversion of lactate to glucose (25). Next, we measured the glucose content in canine hepatocytes following treatment with taurine and betaine. Firstly, canine liver cells were pretreated with taurine or betaine, followed by switching to a gluconeogenesis-induction medium (glucose-free DMEM, supplemented with 20 mM sodium lactate as substrates). The culture medium was collected after 6 h. The newly synthesized glucose secreted into the medium was quantified using a glucose assay kit and normalized to total cellular protein.

**Figure 3:**
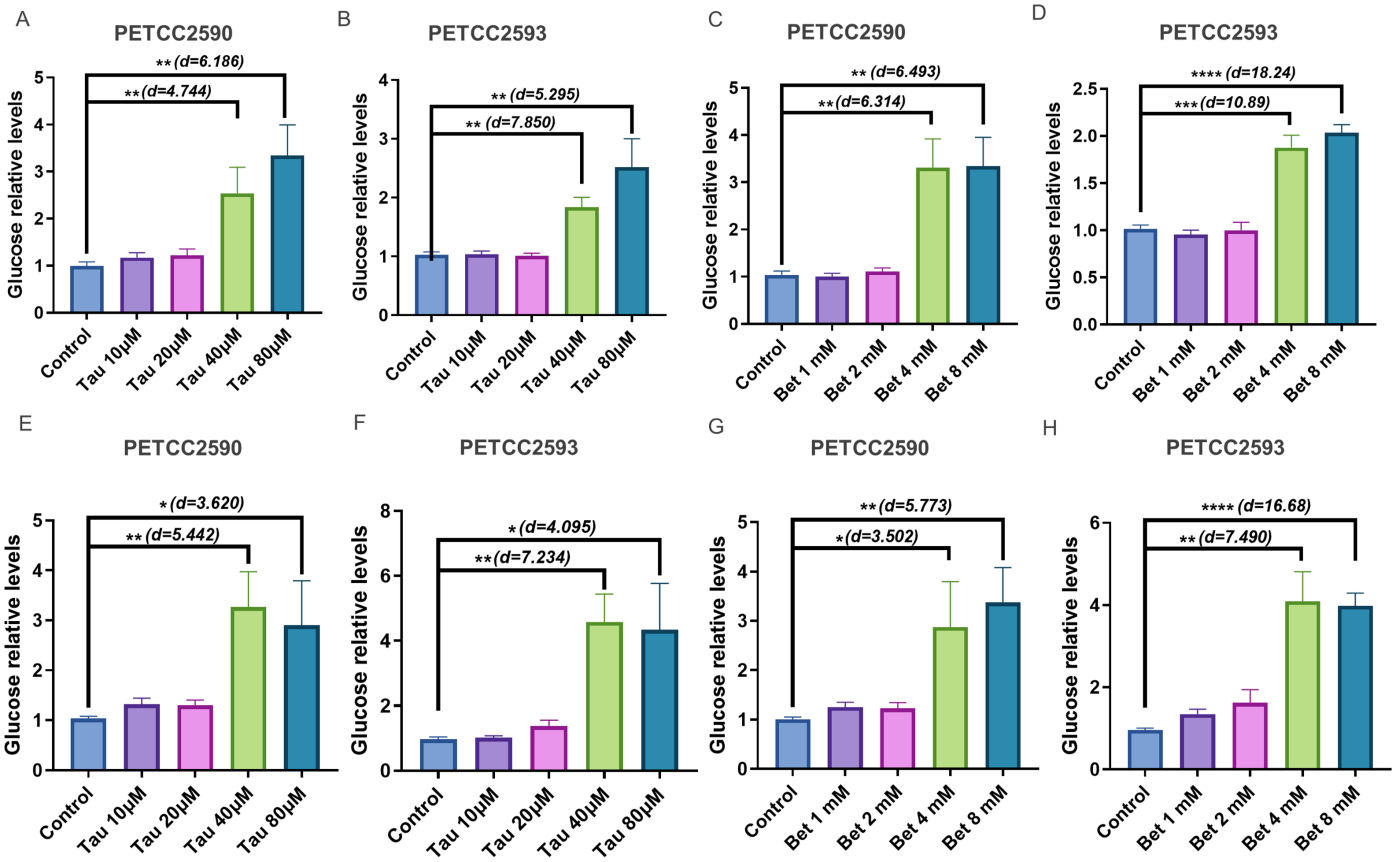
Taurine and betaine increase glucose levels in canine hepatocytes. Canine hepatocytes were treated with varying concentrations of taurine **(A,B)**, betaine **(C,D)** for 48 h in gluconeogenesis-induction medium, and the glucose levels was measured. Canine hepatocytes were treated directly with varying concentrations of taurine **(E,F)**, betaine **(G,H)** for 48 h in normal medium, and the glucose levels was measured. Values are means ± SD from *n* = 3 independent experiments. Statistical differences were determined by Student’s *t* test. * *p* < 0.05, ** *p* < 0.01, *** *p* < 0.001, **** *p* < 0.0001. NS, no significance. Effect sizes (Cohen’s d) for significant comparisons are provided in figure.

The results showed that 40uM (PETCC2590: Student’s *t*-test, df = 4, *p* = 0.009, Cohen’s d = 4.744) (PETCC2593: Student’s *t*-test, df = 4, *p* = 0.0014, Cohen’s d = 7.850) and 80uM (PETCC2590: Student’s *t*-test, df = 4, *p* = 0.0035, Cohen’s d = 6.186) (PETCC2593: Student’s *t*-test, df = 4, *p* = 0.0061, Cohen’s d = 5.295) of taurine increased the rate of glucose production from lactate in both cells compared to the control group (Figures 3A,B). Similarly, 4 mM (PETCC2590: Student’s *t*-test, df = 4, *p* = 0.0032, Cohen’s d = 6.314) (PETCC2593: Student’s *t*-test, df = 4, *p* = 0.0004, Cohen’s d = 10.89) and 8 mM (PETCC2590: Student’s *t*-test, df = 4, *p* = 0.0029, Cohen’s d = 6.493) (PETCC2593: Student’s *t*-test, df = 4, *p* < 0.0001, Cohen’s d = 18.24) treatment with betaine also achieved the same effect (Figures 3C,D). Secondly, we also directly measured the glucose content in the canine liver cells after they were treated with taurine and betaine. The results indicated that treatment with 40 μM (PETCC2590: Student’s *t*-test, df = 4, *p* = 0.0055, Cohen’s d = 5.442) (PETCC2593: Student’s *t*-test, df = 4, *p* = 0.0019, Cohen’s d = 7.234) and 80 μM (PETCC2590: Student’s *t*-test, df = 4, *p* = 0.0224, Cohen’s d = 3.620) (PETCC2593: Student’s *t*-test, df = 4, *p* = 0.0149, Cohen’s d = 4.085) taurine significantly increased glucose levels in the canine hepatocytes (Figures 3E,F). Similarly, treatment with 4 mM (PETCC2590: Student’s *t*-test, df = 4, *p* = 0.0248, Cohen’s d = 3.502) (PETCC2593: Student’s *t*-test, df = 4, *p* = 0.0017, Cohen’s d = 7.490) and 8 mM (PETCC2590: Student’s *t*-test, df = 4, *p* = 0.0045, Cohen’s d = 5.773) (PETCC2593: Student’s *t*-test, df = 4, *p* < 0.0001, Cohen’s d = 16.68) betaine also enhanced glucose content in these cells (Figures 3G,H). This new functional data directly confirms that the observed increase in intracellular glucose is, at least in part, attributable to enhanced gluconeogenic flux.

### Taurine and betaine upregulate gluconeogenesis-related gene expression in canine hepatocytes

**Figure 4:**
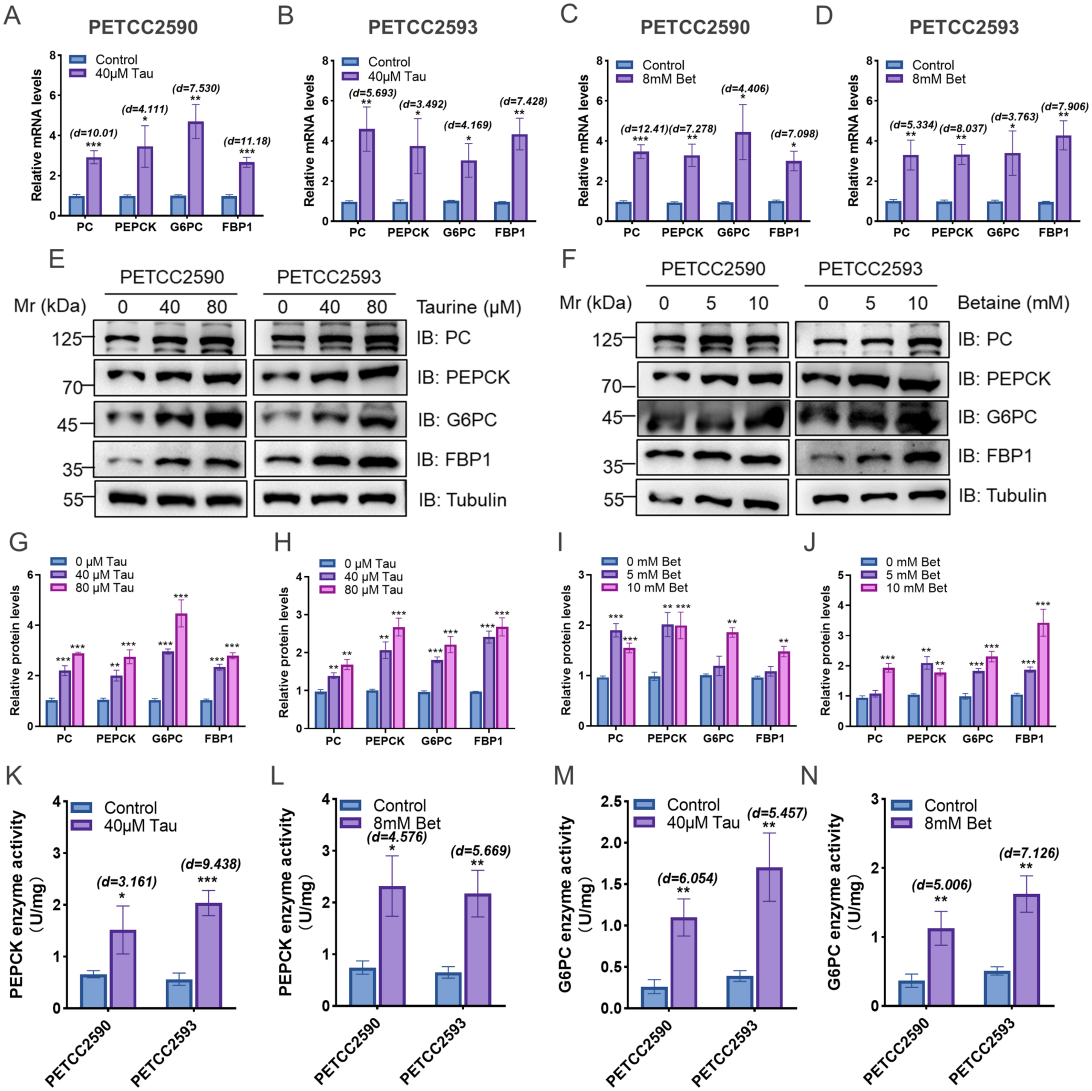
Taurine and betaine upregulate gluconeogenesis-related gene expression in canine hepatocytes. Canine hepatocytes were treated with varying concentrations of taurine, betaine for 48 h, and the gluconeogenesis-related gene mRNA **(A–D)** and protein **(E–J)** expression was measured. The enzymatic activities of PEPCK **(K,L)** and G6PC **(M,N)** was measured. Values are means ± SD from *n* = 3 independent experiments. Statistical differences were determined by Student’s *t* test. * *p* < 0.05, ** *p* < 0.01, *** *p* < 0.001, **** *p* < 0.0001. NS, no significance. Effect sizes (Cohen’s d) for significant comparisons are provided in figure.

We next examined whether taurine and betaine regulate the expression of key gluconeogenic genes in canine hepatocytes. we found that the addition of 40 μM taurine or 8 mM betaine can upregulate the mRNA expression levels of gluconeogenesis-related genes, including PC, PEPCK, G6PC, and FBP1 (Figures 4A–D). In PETCC2590 cells, treatment with 40 μM taurine significantly increased mRNA levels of PC (Student’s *t*-test, df = 4, *p* = 0.0006, Cohen’s d = 10.01), PEPCK (Student’s *t*-test, df = 4, *p* = 0.0147, Cohen’s d = 4.111), G6PC (Student’s *t*-test, df = 4, *p* = 0.0017, Cohen’s d = 7.530), and FBP1 (Student’s *t*-test, df = 4, *p* = 0.0003, Cohen’s d = 11.18). In PETCC2593 cells, similar increases were observed following 40 μM taurine treatment (PC: Student’s *t*-test, df = 4, *p* = 0.0047, Cohen’s d = 5.693; PEPCK: Student’s *t*-test, df = 4, *p* = 0.0251, Cohen’s d = 3.492; G6PC: Student’s *t*-test, df = 4, *p* = 0.0140, Cohen’s d = 4.169; FBP1: Student’s *t*-test, df = 4, *p* = 0.0018, Cohen’s d = 7.428). Treatment with 8 mM betaine also significantly upregulated all four genes in PETCC2590 cells (PC: Student’s *t*-test, df = 4, *p* = 0.0002, Cohen’s d = 12.41; PEPCK: Student’s *t*-test, df = 4, *p* = 0.0019, Cohen’s d = 7.278; G6PC: Student’s *t*-test, df = 4, *p* = 0.0116, Cohen’s d = 4.406; FBP1: Student’s *t*-test, df = 4, *p* = 0.0021, Cohen’s d = 7.098) and in PETCC2593 cells (PC: Student’s *t*-test, df = 4, *p* = 0.0060, Cohen’s d = 5.334; PEPCK: Student’s *t*-test, df = 4, *p* = 0.0013, Cohen’s d = 8.037; G6PC: Student’s *t*-test, df = 4, *p* = 0.0197, Cohen’s d = 3.763; FBP1: Student’s *t*-test, df = 4, *p* = 0.0013, Cohen’s d = 7.906).

Consistent with the mRNA changes, Western blot analysis revealed that taurine and betaine increased protein expression of PC, PEPCK, FBP1, and G6PC in a dose-dependent manner (Figures 4E–J). In PETCC2590 cells, treatment with 40 μM and 80 μM taurine progressively enhanced protein levels of all four enzymes. Quantification showed that 80 μM taurine significantly increased PEPCK expression compared to control. Similarly, betaine treatment (5 mM and 10 mM) dose-dependently upregulated these proteins, with 10 mM betaine significantly elevating G6PC expression. Comparable trends were observed in PETCC2593 cells.

Correspondingly, to determine whether the observed upregulation of gluconeogenic genes translated into functional changes, we measured the enzymatic activities of PEPCK and G6PC. In PETCC2590 cells, treatment with 40 μM taurine significantly increased PEPCK activity (Student’s *t*-test, df = 4, *p* = 0.0342, Cohen’s d = 3.161; Figure 4K) and G6PC activity (Student’s *t*-test, df = 4, *p* = 0.0038, Cohen’s d = 6.054; Figure 4M). In PETCC2593 cells, taurine similarly enhanced PEPCK (Student’s *t*-test, df = 4, *p* = 0.0007, Cohen’s d = 9.438) and G6PC (Student’s *t*-test, df = 4, *p* = 0.0055, Cohen’s d = 5.457) activities. Betaine treatment (8 mM) also significantly increased PEPCK activity in both cell lines (PETCC2590: Student’s *t*-test, df = 4, *p* = 0.0102, Cohen’s d = 4.576; PETCC2593: Student’s *t*-test, df = 4, *p* = 0.0048, Cohen’s d = 5.669; Figure 4L) and enhanced G6PC activity (PETCC2590: Student’s *t*-test, df = 4, *p* = 0.0075, Cohen’s d = 5.006; PETCC2593: Student’s *t*-test, df = 4, *p* = 0.0021, Cohen’s d = 7.126; Figure 4N). These functional data collectively demonstrate that taurine and betaine not only upregulate gluconeogenic gene expression but also enhance the catalytic capacity of key rate-limiting enzymes in the gluconeogenic pathway.

## Discussion

Betaine and taurine are functionally important nutrients that regulate gluconeogenesis through multiple pathways across species. Betaine, a key methyl donor, modulates gluconeogenesis primarily by regulating core gluconeogenic gene expression and alleviating metabolic stress. Previous study demonstrated betaine attenuated alcoholic fatty liver by suppressing DGAT1, DGAT2, SREBP-1c, FAS, SREBP-2 and HMG-CoA reductase while upregulating PGC-1α (26). Moreover, in finishing pigs, betaine supplementation downregulates proteins related to glycolysis, glycogenolysis and glucagon signaling pathway in skeletal muscle, indirectly altering glucose flux and potentially regulating gluconeogenic substrate supply (18). Taurine promotes muscle recovery by alleviating oxidative stress and potentially regulating hepatic metabolic pathways to accelerate lactate clearance. Current evidence suggests betaine regulates gluconeogenesis through epigenetic modification and transcription factor-mediated pathways, while taurine may act through interactions with betaine metabolism and insulin signaling, though synergistic mechanisms require further exploration.

The accumulation of lactate is typically an important physiological indicator following intense exercise, excessive lactate not only leads to muscle fatigue but may also result in a decline in athletic performance (27). Therefore, effectively clearing lactate has become a crucial aspect of improving recovery strategies in exercise. Numerous studies have demonstrated that taurine can reduce lactate levels in the blood of humans and mice following exercise (9, 28), however, its regulatory effects in canines after exercise have not been reported. This finding provides a new perspective on post-exercise recovery in canines and offers potential clues for future research directions.

In this study, we propose that by enhancing hepatic gluconeogenic flux, taurine and betaine supplementation could accelerate the clearance of blood lactate following exercise. This, in turn, would facilitate faster restoration of acid–base balance and glucose homeostasis, indirectly contributing to the alleviation of muscular fatigue and the improvement of recovery. We observed that treatment with taurine and betaine not only reduced lactate levels but also significantly increased the levels of phosphoenolpyruvate and glucose in hepatocytes. Phosphoenolpyruvate is a key intermediate in the gluconeogenesis process, facilitating the conversion of lactate into glucose. Thus, taurine and betaine may promote glucose synthesis by enhancing the generation of critical metabolic intermediates.

It is worth noting that although our findings demonstrate enhanced gluconeogenic flux following taurine supplementation, an alternative or complementary mechanism may involve the regulation of endogenous taurine biosynthesis. As reviewed by Stipanuk, cysteine metabolism via CDO produces cysteine sulfinate, a substrate at a critical branch point (29). One pathway leads to taurine synthesis, while the other leads to the generation of pyruvate, a key gluconeogenic precursor. It is therefore plausible that exogenous taurine provision could downregulate its own biosynthesis, redirecting metabolic flux at this node towards increased pyruvate availability. This elevated pyruvate pool could, in turn, serve as a substrate for enhanced gluconeogenesis, consistent with our functional data. This is not only vital for energy replenishment in canines but may also play an important role in the post-exercise recovery process. Future research could further explore the specific molecular pathways underlying these metabolic regulatory mechanisms to deepen our understanding of canine exercise physiology.

Although our study demonstrates that taurine and betaine enhance gluconeogenic gene expression and enzyme activity, the upstream signaling pathways mediating these effects remain to be elucidated. Based on previous literature, several candidate pathways warrant consideration. AMP-activated protein kinase (AMPK) serves as a master energy sensor and is known to regulate hepatic gluconeogenesis through phosphorylation of CRTC2 and FOXO1, thereby modulating the expression of key gluconeogenic enzymes such as PEPCK and G6PC (30). Interestingly, both taurine and betaine have been implicated in AMPK modulation in other metabolic contexts (31, 32). Additionally, peroxisome proliferator-activated receptor gamma coactivator 1-alpha (PGC-1α) acts as a transcriptional coactivator coordinating the expression of gluconeogenic genes, and its activity is tightly regulated by insulin signaling via the PI3K-Akt-FOXO1 axis (33). Betaine has been shown to influence PGC-1α expression in hepatic tissues (34), while taurine may modulate insulin sensitivity and Akt phosphorylation (35). Therefore, it is plausible that taurine and betaine may act through these interconnected pathways to promote gluconeogenesis in canine hepatocytes. Future studies employing pathway-specific inhibitors or gene silencing approaches are warranted to experimentally validate the involvement of AMPK, PGC-1α, and insulin signaling in the regulatory effects observed in this study.

Overall, this study reveals the potential regulatory effects of taurine and betaine on lactate metabolism and gluconeogenesis in canine hepatocytes, providing new insights for recovery strategies in canines after exercise. As research into canine exercise physiology progresses, these findings may offer new directions for improving athletic performance and health management. It is important to note that this study is deliberately mechanistic and focused on the hepatic lobe of the lactate-glucose axis. While our findings provide strong cellular evidence for enhanced hepatic gluconeogenesis, they do not directly prove efficacy in improving exercise performance in whole animals. This represents a necessary and logical first step in target validation. Future research should therefore prioritize *in vivo* supplementation trials in exercising dogs to validate these mechanistic findings, measuring critical outcome variables such as post-exercise blood lactate clearance rates, time to recovery, and indicators of muscle fatigue. Furthermore, experimental investigations employing molecular approaches (e.g., inhibitor studies, gene knockdown) are needed to delineate the upstream signaling pathways—such as AMPK, PGC-1α, and insulin signaling—that may mediate the gluconeogenic effects of taurine and betaine. Such investigations are essential to translate our *in vitro* findings into practical recovery strategies for canines.

## Data Availability

The original contributions presented in the study are included in the article/supplementary material, further inquiries can be directed to the corresponding authors.

## References

[1] McKenzie BA Chen FL. Assessment and management of declining physical function in aging dogs. Top Companion Anim Med. (2022) 51:100732. doi: 10.1016/j.tcam.2022.100732, 36273752

[2] Soderlund EE Kyrolainen H Laitinen-Vapaavuori OM Hyytiainen HK. Proposed protocol for field testing of endurance fitness of young Labrador retrievers. Methods Protoc. (2023) 6:61. doi: 10.3390/mps6040061, 37489428 PMC10366876

[3] Timlin CL Dickerson SM McCracken F Skaggs PM Fowler JW Amundson LA . Gait, skin and coat, and plasma cytokine changes in response to exercise and trace mineral source. J Anim Sci. (2026) 104:skaf361. doi: 10.1093/jas/skaf361, 41124031 PMC12923159

[4] Erjavec V Vovk T Nemec Svete A. The effect of two acute bouts of exercise on oxidative stress, hematological, and biochemical parameters, and rectal temperature in trained canicross dogs. Front Vet Sci. (2022) 9:767482. doi: 10.3389/fvets.2022.767482, 35359677 PMC8962953

[5] Zanghi BM Robbins PJ Ramos MT Otto CM. Working dogs drinking a nutrient-enriched water maintain cooler body temperature and improved pulse rate recovery after exercise. Front Vet Sci. (2018) 5:202. doi: 10.3389/fvets.2018.00202, 30211176 PMC6121105

[6] Ferraz GC Sgarbiero T Carvalho JRG Almeida MLM Pereira GT Funnicelli MIG . Predicting maximal lactate steady state from lactate thresholds determined using methods based on an incremental exercise test in beagle dogs: a study using univariate and multivariate approaches. Res Vet Sci. (2022) 152:289–99. doi: 10.1016/j.rvsc.2022.08.020, 36081252

[7] Oharomari LK Garcia NF Freitas EC Jordao Junior AA Ovidio PP Maia AR . Exercise training and taurine supplementation reduce oxidative stress and prevent endothelium dysfunction in rats fed a highly palatable diet. Life Sci. (2015) 139:91–6. doi: 10.1016/j.lfs.2015.08.015, 26316449

[8] Kurtz JA VanDusseldorp TA Doyle JA Otis JS. Taurine in sports and exercise. J Int Soc Sports Nutr. (2021) 18:39. doi: 10.1186/s12970-021-00438-0, 34039357 PMC8152067

[9] Akalp K Vatansever Ş Sönmez GT. Effects of acute taurine consumption on single bout of muscular endurance resistance exercise performance and recovery in resistance trained young male adults. Biomed Hum Kinet. (2023) 15:74–82. doi: 10.2478/bhk-2023-0010

[10] Galan BS Carvalho FG Santos PC Gobbi RB Kalva-Filho CA Papoti M . Effects of taurine on markers of muscle damage, inflammatory response and physical performance in triathletes. J Sports Med Phys Fitness. (2018) 58:1318–24. doi: 10.23736/S0022-4707.17.07497-7, 28745470

[11] Chen Q Li Z Pinho RA Gupta RC Ugbolue UC Thirupathi A . The dose response of taurine on aerobic and strength exercises: a systematic review. Front Physiol. (2021) 12:700352. doi: 10.3389/fphys.2021.700352, 34497536 PMC8419774

[12] Elhussiny MZ Tran PV Wang Y Ouchi Y Haraguchi S Gilbert ER . Intracerebroventricular injection taurine changes free amino acid concentrations in the brain and plasma in chicks. Amino Acids. (2023) 55:183–92. doi: 10.1007/s00726-022-03216-7, 36436082

[13] Zawieja E Machek S Zanchi NE Cholewa J Wozniewicz M. Effects of chronic betaine supplementation on exercise performance: systematic review and meta-analysis. J Sports Sci. (2024) 42:2131–44. doi: 10.1080/02640414.2024.2423578, 39514262

[14] Nobari H Cholewa JM Perez-Gomez J Castillo-Rodriguez A. Effects of 14-weeks betaine supplementation on pro-inflammatory cytokines and hematology status in professional youth soccer players during a competition season: a double blind, randomized, placebo-controlled trial. J Int Soc Sports Nutr. (2021) 18:42. doi: 10.1186/s12970-021-00441-5, 34090451 PMC8180114

[15] Zawieja E Chmurzynska A. Betaine and aging: a narrative review of findings, possible mechanisms, research perspectives, and practical recommendations. Ageing Res Rev. (2025) 104:102634. doi: 10.1016/j.arr.2024.102634, 39647584

[16] Di Credico A Gaggi G Izzicupo P Vitucci D Buono P Di Baldassarre A . Betaine treatment prevents TNF-α-mediated muscle atrophy by restoring Total protein synthesis rate and morphology in cultured Myotubes. J Histochem Cytochem. (2023) 71:199–209. doi: 10.1369/00221554231165326, 37013268 PMC10149894

[17] Buonaiuto G Federiconi A Vecchiato CG Benini E Mordenti AL. Betaine dietary supplementation: healthy aspects in human and animal nutrition. Antioxidants. (2025) 14:771. doi: 10.3390/antiox14070771, 40722875 PMC12291949

[18] Cai D Jia Y Song H Sui S Lu J Jiang Z . Betaine supplementation in maternal diet modulates the epigenetic regulation of hepatic gluconeogenic genes in neonatal piglets. PLoS One. (2014) 9:e105504. doi: 10.1371/journal.pone.0105504, 25153319 PMC4143294

[19] Zhang Q Bertics SJ Luchini ND White HM. The effect of increasing concentrations of dl-methionine and 2-hydroxy-4-(methylthio) butanoic acid on hepatic genes controlling methionine regeneration and gluconeogenesis. J Dairy Sci. (2016) 99:8451–60. doi: 10.3168/jds.2016-11312, 27474977

[20] Seidel U Eberhardt K Wiebel M Luersen K Ipharraguerre IR Haegele FA . Stearidonic acid improves eicosapentaenoic acid status: studies in humans and cultured hepatocytes. Front Nutr. (2024) 11:1359958. doi: 10.3389/fnut.2024.1359958, 38974810 PMC11225816

[21] Lombardini JB. Increased phosphorylation of specific rat cardiac and retinal proteins in taurine-depleted animals: isolation and identification of the phosphoproteins. Adv Exp Med Biol. (1998) 442:441–7. doi: 10.1007/978-1-4899-0117-0_54, 9635061

[22] Komine S Miyazaki T Ishikura K Matsui T Miyoshi T Ra SG . Taurine supplementation enhances endurance capacity by delaying blood glucose decline during prolonged exercise in rats. Amino Acids. (2022) 54:251–60. doi: 10.1007/s00726-021-03110-8, 35122528 PMC8894168

[23] Song Q Kobayashi S Kataoka Y Oda H. Direct molecular action of taurine on hepatic gene expression associated with the amelioration of hypercholesterolemia in rats. Antioxidants (Basel). (2024) 13:990. doi: 10.3390/antiox13080990, 39199235 PMC11351134

[24] Wang Z Dong C. Gluconeogenesis in cancer: function and regulation of PEPCK, FBPase, and G6Pase. Trends Cancer. (2019) 5:30–45. doi: 10.1016/j.trecan.2018.11.003, 30616754

[25] Legouis D Faivre A Cippa PE de Seigneux S. Renal gluconeogenesis: an underestimated role of the kidney in systemic glucose metabolism. Nephrol Dial Transplant. (2022) 37:1417–25. doi: 10.1093/ndt/gfaa302, 33247734

[26] Yang W Huang L Gao J Wen S Tai Y Chen M . Betaine attenuates chronic alcohol-induced fatty liver by broadly regulating hepatic lipid metabolism. Mol Med Rep. (2017) 16:5225–34. doi: 10.3892/mmr.2017.7295, 28849079 PMC5647077

[27] Lee S Choi Y Jeong E Park J Kim J Tanaka M . Physiological significance of elevated levels of lactate by exercise training in the brain and body. J Biosci Bioeng. (2023) 135:167–75. doi: 10.1016/j.jbiosc.2022.12.001, 36681523

[28] Li X Huo L Wang L Zhang W. Dose-response relationship of taurine on endurance cycling performance under hot and humid conditions. Front Nutr. (2025) 12:1632131. doi: 10.3389/fnut.2025.1632131, 41158650 PMC12554441

[29] Stipanuk MH Dominy JE Jr Lee JI Coloso RM. Mammalian cysteine metabolism: new insights into regulation of cysteine metabolism. J Nutr. (2006) 136:1652S–9S. doi: 10.1093/jn/136.6.1652s, 16702335

[30] Zhang Y Chen J Zeng Y Huang D Xu Q. Involvement of AMPK activation in the inhibition of hepatic gluconeogenesis by *Ficus carica* leaf extract in diabetic mice and HepG2 cells. Biomed Pharmacother. (2019) 109:188–94. doi: 10.1016/j.biopha.2018.10.077, 30396076

[31] Chen W Zhang X Xu M Jiang L Zhou M Liu W . Betaine prevented high-fat diet-induced NAFLD by regulating the FGF10/AMPK signaling pathway in ApoE(−/−) mice. Eur J Nutr. (2021) 60:1655–68. doi: 10.1007/s00394-020-02362-6, 32808060

[32] Karikkakkavil Prakashan AD Muthukumar SP Martin A. Taurine activates SIRT1/AMPK/FOXO1 signaling pathways to favorably regulate lipid metabolism in C57BL6 obese mice. Mol Nutr Food Res. (2024) 68:e2200660. doi: 10.1002/mnfr.202200660, 38549461

[33] Yoon JC Puigserver P Chen G Donovan J Wu Z Rhee J . Control of hepatic gluconeogenesis through the transcriptional coactivator PGC-1. Nature. (2001) 413:131–8. doi: 10.1038/35093050, 11557972

[34] Wu C Zhang W Liu W Tang Z Peng S Fu L . Cystathionine gamma-lyase downregulation promotes liver injury and necroptosis through reprogramming of methionine cycle. Redox Rep. (2025) 30:2531650. doi: 10.1080/13510002.2025.2531650 40717553 PMC12305881

[35] Zhu X Li J Wang H Gasior FM Lee C Lin S . TAT delivery of a PTEN peptide inhibitor has direct cardioprotective effects and improves outcomes in rodent models of cardiac arrest. Am J Physiol Heart Circ Physiol. (2021) 320:H2034–43. doi: 10.1152/ajpheart.00513.2020, 33834871 PMC8163648

